# Understanding the Associations Between Attachment Insecurity, Emotional Flooding, and Conflict Behaviors in Prenatal Couples

**DOI:** 10.1111/jmft.70142

**Published:** 2026-05-06

**Authors:** Sean D. Morgan, Erica M. Woodin

**Affiliations:** ^1^ Department of Psychology University of Victoria Victoria British Columbia Canada

**Keywords:** attachment style, couple conflict, emotional flooding, transition to parenthood

## Abstract

The transition to parenthood is a period of heightened vulnerability for many couples, often marked by increased conflict. Attachment insecurity is a known risk factor, linked to dysfunctional conflict dynamics in couples more broadly. Yet, the emotional processes driving these patterns remain underexplored, especially from a dyadic perspective. This study examined *emotional flooding*—a form of dyadic emotion dysregulation—as a potential intermediary variable linking attachment to conflict behaviors. Ninety‐six mixed‐sex couples completed questionnaires on attachment and emotional flooding before engaging in observationally coded conflict discussions during the third trimester of pregnancy. Actor partner interdependence models extended to mediation (APIMeM) revealed that men's attachment anxiety was positively associated with observed conflict withdrawal indirectly through their own emotional flooding. Women's attachment anxiety was positively associated with observed conflict hostility through their own emotional flooding. Findings highlight emotional flooding as a pathway through which attachment shapes mixed‐sex couples' vulnerability to gendered conflict patterns.

It has been theorized that emotions play a crucial role in understanding how the attachment system of romantic partners may predispose them to certain conflict behaviors. For example, partners with insecure attachment often show heightened appraisals to threats, perceiving others as being unavailable and unresponsive to their needs (e.g., failing to provide protection and support; Ein‐Dor et al. 2011). Despite evidence that attachment insecurity contributes to poorer couple functioning in general populations (Feeney and Fitzgerald 2019), few studies have directly examined these processes in expectant couples, even though conflict during this stage often intensifies (Kluwer 2010), has downstream implications for coparenting and child adjustment (Cowan and Cowan 2000), and is associated with poor adaptation between partners after birth (Heinicke and Guthrie 1996).

Evidence suggests that emotion dysregulation peaks during pregnancy and declines after childbirth (Zhou et al. 2025), highlighting this stage as a period of particular vulnerability. Understanding the link between emotional reactivity and couple conflict behaviors could help clarify mechanisms that underlie prominent theoretical frameworks of the transition to parenthood. For example, both diathesis–stress models (Simpson and Rholes 2012) and other transition to parenthood frameworks (Kluwer 2010) emphasize that stress can undermine couple functioning; yet, they often allude to, rather than directly test, the emotional processes that may connect vulnerability factors (e.g., attachment insecurity) to outcomes (e.g., couple conflict behaviors).

We explored one potential explanation for this attachment–behavior association, *emotional flooding*, which taps into both the intra‐ and interpersonal nature of dysregulated emotions within dyads. Emotional flooding is a response to partners' negative behavior during conflict, explained as an emotional overwhelm during the interaction (Gottman 1993), while also being understood as a dispositional vulnerability (Malik et al. 2020). Conceptualizing emotional flooding as an explanatory variable may help clarify why conflict is most likely to escalate, with flooding serving as a warning signal that emotion regulation capacities have been exceeded.

## 1 Adult Attachment and Couple Conflict Behaviors

Adult attachment is often described along two continuous dimensions: attachment anxiety and avoidance (Fraley and Roisman 2019). Anxious attachment is characterized by a fear of rejection by one's romantic partner, leading to a hyperactivation of the attachment system and downstream reassurance seeking from one's romantic partner. Avoidance is characterized by a fear of intimacy and discomfort in romantic relationships, leading to a deactivation of the attachment system and a suppression of negative feelings (Mikulincer and Shaver 2007). During relationship conflict, these core attachment fears (i.e., fear of rejection and fear of closeness) may be triggered, activating emotion regulation processes that can contribute to heightened distress.

Anxiously attached individuals tend to report greater distress (Campbell et al. 2005) and harbor anger (Mikulincer 1998), patterns that have been linked to broader difficulties in emotion regulation (Gentzler et al. 2010). Although avoidantly attached individuals rely on deactivating strategies such as emotional suppression, these strategies do not eliminate emotional arousal and are associated with heightened physiological reactivity (Powers et al. 2006). As such, suppression may become less effective under stress, potentially resulting in increased emotional overwhelm. Consistent with this, insecure attachment has been associated with more maladaptive conflict behaviors (e.g., demandingness and withdrawal) and fewer constructive behaviors (e.g., self‐disclosure and problem‐solving) compared to secure counterparts (Domingue & Mollen, 2009).

Although there is an intuitive link between attachment anxiety and demanding behaviors (due to hyperactivation and reassurance‐seeking tendencies; Mikulincer and Shaver 2007), and between attachment avoidance and withdrawal behaviors (reflecting deactivation and suppression; Mikulincer and Shaver 2007), empirical findings have been mixed. Evidence from non‐dyadic research, often relying on undergraduate samples, suggests that both attachment dimensions may be associated with multiple forms of maladaptive conflict behavior. For example, Bonache et al. (2019) found that attachment avoidance was related to withdrawal, whereas anxiety was related to both conflict engagement and withdrawal. Similarly, Shi (2003) found that attachment avoidance was associated with both dominating behavior and withdrawal, whereas anxiety was associated with dominating behavior.

Findings from dyadic samples further underscore this variability. Sierau and Herzberg (2012) found that one's own attachment anxiety and avoidance predicted conflict engagement and withdrawal (actor effects), with no partner effects observed. Similarly, Gonzales et al. (2019) reported that both attachment dimensions were associated with engagement and withdrawal, although withdrawal was more strongly associated with attachment avoidance and engagement was more strongly associated with attachment anxiety. Together, these findings suggest that attachment insecurity may relate broadly to multiple forms of maladaptive conflict behavior, while still reflecting tendencies for attachment anxiety to be associated with conflict engagement and attachment avoidance to withdrawal.

Understanding the attachment–conflict behavior association might be further complicated by gender dynamics. Some research has identified gendered demand–withdraw patterns in mixed‐sex couples, with women more likely to engage in demanding behaviors and men more likely to withdraw (Baucom et al. 2010). Importantly, these patterns appear linked, in part, to attachment insecurity. For example, in a community sample of 64 couples, women's attachment anxiety and men's attachment avoidance were associated with women's demands and men's withdrawal (Rodriguez 2000). These tendencies may reflect the interpersonal expression of attachment‐related regulation strategies, where hyperactivating strategies associated with anxiety manifest as pursuing or demanding behaviors, and deactivating strategies associated with avoidance manifest as withdrawal.

At the same time, evidence for gender differences is mixed. Shi (2003) found only minimal gender differences in conflict avoidance, suggesting that these patterns are not universal. Other research indicates that context, such as conflict initiator, may shape these dynamics. For instance, Seedall (2024) found that women higher in attachment anxiety displayed more demanding behaviors, whereas men higher in attachment anxiety displayed fewer demands, particularly when the conflict topic was initiated by the man. In contrast, men were generally more likely to withdraw during conflicts centered on women's topics, regardless of attachment orientation. Taken together, these findings suggest that gender differences in attachment–conflict behavior may exist, but could also be shaped by contextual and interpersonal factors. Examining the underlying emotional processes that disrupt regulation during conflict may help clarify the intermediary variables through which attachment insecurity translates into patterns of conflict behavior and whether gender effects emerge.

## 2 Attachment and Emotional Flooding

Understanding how attachment insecurity relates to difficulties regulating emotions during couple conflict has typically been explored using intrapersonal measures (e.g., the Difficulties in Emotion Regulation Scale [DERS]; Gratz and Roemer 2004) that tap into one's difficulty engaging in goal‐directed behavior, having limited access to strategies, and lacking emotional clarity/awareness. Indeed, deficits in emotion regulation skills predict a host of negative outcomes (e.g., sexual, psychological, and physical aggression; Halmos et al. 2021; Lee et al. 2020). This intrapersonal conceptualization, however, often neglects the context in which the dysregulation is occurring (e.g., during couple conflict). Emotion regulation is a deeply social process (Rimé 2010), shaping how individuals decode partner behaviors, regulate their own emotions, and enact responses during interactions (Dixon‐Gordon et al. 2015).

Attachment insecurity may increase vulnerability to experiencing intense emotional reactions during conflict. Anxiously attached individuals tend to perceive partner negativity as threatening and overwhelming, whereas avoidantly attached individuals may perceive partner demands as intrusive and disorganizing (Mikulincer and Shaver 2007). Emotional flooding, the subjective sense of being overwhelmed by a partner's negative affect, reflects this interplay between internal experience and relational context. Flooded individuals often perceive conflict cues as more threatening than they are, may distort situational information, and enter a heightened state of negative attribution that is difficult to modify (Del Vecchio et al. 2016). Both forms of attachment insecurity may predispose individuals to flooding. Anxiety may be associated through hyperarousal and amplified negative affect, whereas avoidance may be associated through failed suppression attempts that accompany increased physiological reactivity (Mikulincer and Shaver 2016; Powers et al. 2006).

Potential gender differences have been observed in these dynamics. Gottman (1993) suggested that men may be more easily flooded by negative affect, a finding partially supported by Foran et al. (2020), who observed slightly higher mean flooding in men, though Malik et al. (2020) found no gender differences. These inconsistencies may reflect individual or contextual differences, highlighting the importance of examining flooding not only in relation to individual factors (e.g., attachment insecurity) but also as an interpersonal process that shapes conflict dynamics. Preliminary work in this area also suggests that gender differences in flooding may emerge under certain conditions, potentially aligning with the modest gender effects observed in the demand–withdraw literature (Seedall 2024).

## 3 Flooding and Conflict Behaviors

Although similar to constructs such as distress intolerance (Zvolensky et al. 2010), emerging evidence conceptualizes flooding as both an individual difference in distress tolerance and an inherently interpersonal phenomenon contingent on a partner's behavior (Malik et al. 2020). When one partner becomes overwhelmed by the other's negative affect, they may respond by withdrawing, escalating, or attempting to end the interaction to manage their distress (Gottman 1993). These responses can manifest as observable conflict behaviors such as hostility, demandingness, or withdrawal. Consistent with this, flooding has been linked to maladaptive dynamics, including demand–withdraw conflict patterns (Biesen et al. 2023) and psychological and physical intimate partner violence (Foran et al. 2020; Malik et al. 2020).

Gottman (1993) hypothesized that conflict escalation often begins with emotional flooding. When experiencing cognitive overwhelm and difficulty self‐regulating, individuals may rely on overlearned behaviors because they are unable to attend to anything but their physiological state. Attempts to escape the situation are often perceived by the partner as unexpected and disorganized, mirroring the subjective experience of flooding. Biesen et al. (2023) found that men's flooding was associated with both woman‐demand/man‐withdraw and man‐demand/woman‐withdraw patterns in 87 heterosexual couples, though these associations were not examined for distinct gender differences, and no partner effects emerged. Similarly, in a sample of 291 heterosexual couples, both men's flooding and women's flooding were positively associated with their own anger expression during conflict, and men's flooding predicted their own withdrawal during conflict discussions initiated by women.

Together, these findings suggest that emotional flooding may be a key mechanism through which insecure attachment translates into hostile or withdrawn conflict behaviors. Although insecurity and emotion dysregulation have been widely studied (Dixon‐Gordon et al. 2015), research explicitly examining flooding remains scarce. By capturing how attachment‐related vulnerabilities unfold in dyadic interactions, flooding provides a potential intermediary variable linking internal emotional processes to specific interpersonal behaviors.

## 4 Prenatal Time Period

The prenatal period provides a unique context for examining attachment, emotional flooding, and conflict behaviors in couples. This life stage involves major transitions, including new responsibilities, role shifts, and anticipated demands of parenthood, which can activate attachment‐related expectations and vulnerabilities (Feeney 2008; McNulty et al. 2021). Research indicates that attachment systems become particularly salient during pregnancy, influencing both individual stress responses and conflict behaviors (Kohn et al. 2012; Simpson et al. 2003).

Gendered differences may also emerge under these conditions. For example, men may be more likely to rely on deactivating strategies under relational stress (e.g., emotional suppression and withdrawal from conflict), whereas women may be more likely to engage in hyperactivating strategies (e.g., increased emotional expression and demands during conflict), particularly in the context of heightened relational threat (STEVEN RHOLES et al. 2014). These differential regulatory tendencies may have implications for the experience of emotional flooding, insofar as deactivating strategies may fail to downregulate physiological arousal under escalating conflict, whereas hyperactivating strategies may amplify perceived threat and emotional intensity. Together, these processes provide a theoretical basis for examining whether attachment‐related emotion regulation and flooding may operate in gender‐specific ways during prenatal couple conflict.

Conflict during pregnancy has implications that extend into the postnatal period. Prenatal negativity can spill over into coparenting and broader family functioning, predicting lower relationship quality, less supporting coparenting, and adjustment difficulties for parents and children (Katz and Gottman 1996; Heinicke and Guthrie 1996; Kuersten‐Hogan et al. 2021; Shapiro et al. 2000). Studying couples in the third trimester captures early risk patterns, providing insight into how attachment‐related emotional processes, including emotional flooding, may shape conflict behaviors before the added stressors of newborn care emerge.

## 5 Current Study

The present study extends research on prenatal couple conflict dynamics by examining emotional flooding as a mechanism linking attachment insecurity to conflict behaviors. Drawing on adult attachment theory (Mikulincer and Shaver 2005), we conceptualized attachment insecurity as a risk factor for emotional flooding, given the maladaptive emotional heuristics associated with insecure attachment (Malik et al. 2020; Mikulincer and Shaver 2007). Flooding, in turn, was expected to be positively associated with more intense conflict behaviors, as cognitive overwhelm during this “fight‐or‐flight” state may trigger reliance on overlearned, maladaptive responses (Gottman 1993). To capture these processes as inherently dyadic, we examined both partners simultaneously, highlighting the interpersonal pathways between attachment, flooding, and conflict behaviors. To our knowledge, this is the first study to test emotional flooding as a potential intermediary variable between attachment insecurity and observed conflict behaviors.

### 5.1 Hypotheses

Emotional flooding (Gottman 1993) may help explain how intrapersonal risk factors (e.g., attachment insecurity) relates to interpersonal difficulties (e.g., couple conflict). Prior research on conflict behavior suggests that gender differences may be observed in the expression of demand and withdrawal behaviors during couple conflict, although findings are mixed (Rodriguez 2000; Seedall 2024). One potential explanation for these patterns is that men and women may differ in how they regulate emotional arousal during conflict, which may in turn influence susceptibility to emotional flooding. From this perspective, emotional flooding may represent an intermediary variable linking attachment‐related regulation tendencies to observable conflict behaviors. Accordingly, and consistent with prior theoretical and empirical work, we tentatively hypothesize that:

Attachment anxiety would be indirectly related to hostility through emotional flooding in women during a laboratory‐based conflict discussion. For example, attachment anxiety would be positively associated with flooding, which would be positively associated with displays of hostility in women.

Attachment avoidance would be indirectly related to withdrawal through emotional flooding in men during a laboratory‐based conflict discussion. For example, attachment avoidance would be positively associated with flooding, which would be positively associated with displays of withdrawal in men.

These gendered hypotheses are grounded in prior observations that hyperactivating tendencies associated with attachment anxiety may align with more demanding behaviors and deactivating tendencies associated with attachment avoidance may align with withdrawal, but we acknowledge that evidence for gender differences remains variable. Consequently, we also examined all pathways from attachment insecurity to conflict behaviors through flooding and explored gender as a potential moderator. Finally, we explored whether emotional flooding might relate to positive conflict behaviors (e.g., problem‐solving, intimacy), although these analyses were considered exploratory, given limited prior research.

## 6 Methods

### 6.1 Participants

Participants were recruited from a mid‐sized Canadian West Coast city through public advertisements, expectant‐parent events, and community programs for first‐time parents. Eligible couples were cohabiting, at least 17 years old, in the third trimester of their first pregnancy, and able to read and write in English. We included participants aged 17 years and older to capture younger couples living independently with their partner and to remain inclusive of early parenthood experiences. Couples were informed that the study aimed to examine relationship functioning during the transition to parenthood. As part of a larger longitudinal project of 100 mixed‐sex couples assessed at the third trimester of pregnancy, 96 couples with observational data available were included in the current study. Although the exact numbers of initially interested participants are unavailable, most non‐participation was due to incomplete responses rather than ineligibility. Two couples were in same‐sex relationships, and two couples' data were lost to technological errors. Because the small number of same‐sex couples precluded meaningful comparisons with mixed‐sex couples, and collapsing across relationship types would risk conflating gender effects and assuming homogeneity, these couples were not included in the analyses.

All procedures were approved by the University of Victoria Research Ethics Board (21‐0422) and an honorarium of $50 was provided to each participant at the prenatal time point. The average age of the participants was 31.98 years (SD = 5.42) for men and 30.00 years (SD = 5.38) for women. Couples reported living together for 4.46 years (SD = 3.23). Most (69.8%) of the sample reported being legally married, with the rest (30.2%) reporting being unmarried cohabitating. The average income was $51,825 (SD = 35,614) for men and $35,259 for women (SD = 25,027). Men reported 14.82 years of education (SD = 2.37) and women reported 15.31 years (SD = 2.31), both corresponding to some university education. In terms of ethnicity, a majority of men (89.6%) and women (87.5%) identified as white, with the remaining participants representing diverse racial and ethnic backgrounds, including Indigenous, Asian (e.g., Chinese, East Indian, Japanese, and Filipina), Latin American, African, and Sikh identities.

### 6.2 Procedure

At the prenatal time point, interested couples contacted the laboratory and were screened by phone. Then, appointments were scheduled for eligible participants to attend the 3‐h research session. Participants provided informed consent prior to data collection. Partners were first separated to fill out a series of self‐report questionnaires. Each couple then engaged in two conflict interactions for 10 min each. These interactions were videotaped and observationally coded by trained research assistants.

To determine a topic for the conflict interactions, partners were given a list of potential topics and asked to indicate on a scale (0‐100) the degree to which they wanted to change each area in their relationship. Example domains included household chores (e.g., cleaning), finances (e.g., paying bills on time), and showing appreciation for others. A researcher then collected the forms and asked each partner to describe their highest‐scoring item. If both partners selected the same topic, one partner discussed their second‐highest rated topic to ensure two distinct discussions. Random assignment was used to determine which partner would go first. Couples conversed on their selected topics for 10 min each. After 10 min, a researcher would enter the room and indicate that the other partner should begin their topic, leading to the next 10‐min discussion.

### 6.3 Measures

#### 6.3.1 Attachment

To assess adult attachment style, participants completed the Experiences in Close Relationships Scale (ECR; Brennan et al. 1998). The 36‐item questionnaire includes 18 items that measure attachment anxiety (e.g., “I'm afraid that I will lose my partner's love”) and 18 items that measure attachment avoidance (e.g., “I find it difficult to allow myself to depend on romantic partners”). Items measuring anxiety and avoidance were separately summed to produce two dimensions of attachment insecurity, with lower scores on both domains indicating greater security. Summed scores are linearly equivalent to mean scores and preserve the dimensional interpretation of the measure (Tabachnick and Fidell 2013). Items were rated on a 7‐point Likert scale ranging from 1 (strongly Disagree) to 7 (strongly Agree). This scale is widely used to measure attachment in couples research and has been applied to studies on emotion dysregulation and negative behaviors (Cheche‐Hoover et al. 2019; Dominigue & Mollen, 2009). Reliability was adequate for both attachment anxiety ($\alpha_{\text{Men}}\;$= 0.86; $\alpha_{\text{Women}}$ = 0.89) and avoidance ($\alpha_{\text{Men}}$ = 0.90; $\alpha_{\text{Women}}$ = 0.87) in our sample.

#### 6.3.2 Flooding

The Intimate Partner Flooding Scale (IPFS; Foran et al. 2020) is a 15‐item questionnaire that assesses emotional flooding or the subjective experience of being overwhelmed and disorganized based on a partner's negative affect during conflict. Participants rate their propensity to become flooded on a 5‐point Likert scale, ranging from 1 (never) to 5 (almost always), with higher scores indicating greater flooding. Example items include “I find my partner's anger to be overwhelming” and “My partner's anger seems to come out of nowhere”. Based on the recommendations of the original scale's author and because several items were highly intercorrelated, only 9 out of the 15 items were retained (Foran & Slep, 2007). This reduced redundancy and produced a more stable and interpretable measure of emotional flooding. This measure is unifactorial, has excellent internal consistency, and has strong concurrent validity with observed negative conflict behaviors (Foran et al. 2020). Cronbach's alpha for the scale was excellent for both men (α = 0.90) and women (α = 0.91) in our sample.

#### 6.3.3 Conflict Behaviors

Recent work highlights the “lump versus split” dilemma, referring to whether behaviors are aggregated into broad categories (“lump”) or kept discrete (“split”; Heyman et al. 2021). Synthesizing multiple studies using the same coding system, Heyman et al. (2021) found support for the “lump” approach, showing that discrete behaviors coalesce into two valence‐based factors (negative and non‐negative across genders).

Biphasic approaches to emotion (e.g., Bradley 2000) conceptualize affect along valence (negative to positive) and arousal (low to high) dimensions. Whereas the “lump” approach has emphasized valence, arousal is also critical, as it differentiates behaviors of the same valence (e.g., contempt vs. irritation) and aligns with high‐intensity codes in pivotal coding schemes (Shapiro and Gottman 2004). Incorporating both valence and arousal thus strengthens the theoretical bridge between conflict coding and emotion science.

To align with the “lump” approach to observational coding and the biphasic model of emotion, couple conflict interactions were coded using the Couples Affect Intensity Rating System (CAIRS; Woodin & Galaugher, 2012). This system follows the meta‐analysis by Woodin (2011), which identified five categories of conflict behaviors distinguished by valence (negative to positive) and intensity (low to high), consistent with emotion research. These five behaviors include hostility, withdrawal, distress, problem‐solving, and intimacy, rated on a Likert scale ranging from 1 (never) to 5 (almost always).

Each category is composed of specific codes. Hostility involves high‐intensity negative behaviors, such as belligerence (provoking the partner), criticism (criticizing a partner's personality or character), and contempt (communicating disrespect). Withdrawal reflects attempts to disengage from discussion, including stonewalling (ceasing to attend to the partner) and avoidance (diverting attention from the conflict topic). Distress captures lower intensity negative affect, including tension (anxious or worried reactions), sadness (passivity, crying, and pouting), and anger (irritation and annoyance). Intimacy reflects efforts to enhance closeness and understanding, whereas problem‐solving refers to constructive communication aimed at resolving issues.

Observational coders received 10 h of training on the CAIRS system. Each conflict interaction was coded in two 5‐min segments using the CAIRS, after which the scores were averaged across the two segments. Given that this coding system has not been formally published, an exploratory factor analysis (EFA) was run to determine the number of factors to retain. Results of Kaiser–Meyer–Olkin (KMO) measures of sampling adequacy (men = 0.71; women = 0.65) and significant Bartlett's tests of sphericity for men (*χ* ^2^(105) = 537.66, *p* < 0.001) and women (*χ* ^2^(105) = 332.95, *p* < 0.001) indicated that data were adequate for EFA models (Tabachnick & Fidell, 2007). Research suggests that KMO values above 0.6 are acceptable for sample sizes under 100 (Shrestha 2021), indicating that the factor structure was suitable despite the modest KMO.

Principal axis factoring was used as the extraction method with oblimin rotation based on the hypothetical correlation between factors. Separate models were run for women and men. The final solutions were determined by examining scree plots with their corresponding eigenvalues (Gorsuch 1990). We also took into consideration (a) an efficient number of loadings (> 0.40) and (b) conceptual interpretability. The EFA indicated that Withdrawal and Distress should be combined into a single factor, consistent with the overlap between avoidance and lower intensity distress behaviors. For example, tension loaded strongly with stonewalling, whereas other codes had low loadings. Consequently, we retained 4 factors for both women and men: Hostility, Withdrawal, Intimacy, and Problem‐Solving. Factor loadings are presented in Table 1. Interrater correlation coefficients (ICCs) ranged from 0.62 to 0.80 for men and from 0.49 to 0.91 for women prior to creating factor‐level composites. At the factor level, ICCs were adequate for hostility (men = 0.843, women = 0.846), withdrawal (men = 0.618, women = 0.767), and intimacy (men = 0.747, women = 0.770). ICCs for problem‐solving were lower (men = 0.547, women = 0.490). As discussed later, this factor was not included in the present analyses. Overall, the reliability of the factors used in our analyses was acceptable, supporting the use of the CAIRS to examine conflict behaviors in this sample.

**Table 1:** Exploratory factor analysis (EFA) results for CAIRS codes.

CAIRS code	Factor 1 (hostility)		Factor 2 (withdrawal)		Factor 3 (intimacy)		Factor 4 (problem‐solving)	
	Men	Women	Men	Women	Men	Women	Men	Women
Belligerence	**0.634**	**0.739**					−0.510	
Criticism	**0.755**	**0.483**						
Contempt	**0.788**	**0.713**	0.446	0.452				
Domineering	**0.645**	**0.569**			−0.592	−0.609		
Defensiveness	**0.708**	**0.413**			−0.428			
Anger	**0.550**	**0.377**						
Stonewalling			**0.509**	**0.536**				
Tension			**0.756**	**0.715**				
Affection					**0.450**	**0.505**		
Empathy					**0.447**	**0.480**		
Humor					**0.576**	**0.566**		
Solution‐Seeking							**0.905**	**0.677**
Engagement							**0.764**	**0.755**

*Note:* Extraction Method: Principal Axis Factoring. Rotation method: Oblimin with Kaiser Normalization. Factor loadings in bold refer to retained factors.

### 6.4 Data Analysis Plan

Actor Partner Interdependence Models extended to Mediation (APIMeM; Ledermann et al. 2011) allow the estimation of a variable's association with one's own scores (e.g., an actor effect) as well as one's partner (e.g., a partner effect). APIMeM consists of predictor variables (e.g., attachment anxiety and avoidance), outcome variables (e.g., conflict behaviors), and mediators (flooding) for each partner. Mediation is determined by examining direct effects, indirect effects, and total effects (Ledermann et al. 2011). APIMeM also considers the interdependence between partners, which accounts for the nestedness of the sample (e.g., romantic partners within relationships).

Given the statistical complexity of APIMeM models, we first tested whether variables were meaningfully different based on gender (known as distinguishability). We compared models that constrained or unconstrained means, variances, and covariances across men and women, with a significant chi‐square test indicating distinguishability. We also used the Comparative Fit Index (CFI; CFI > = 0.96 indicating good model fit; Olsen and Kenny 2006), the Root Mean Square Residual (SRMR; SRMR <= 0.08 indicate adequate model fit; Kenny et al. 2015), and the Root Mean Square Error of Approximation (RMSEA; RMSEA < 0.06 indicate adequate model fit for the data; Olsen and Kenny 2006) to test for model fit. In order to test potential mediating effects, the significance levels of indirect effects for each model were tested using bootstrapping (number of bootstrap draws = 5000). Standardized indirect effects were computed for each of the 5000 bootstrapped subsamples, and a 95% confidence interval was computed.

For parsimony, we also examined dyadic patterns with the *k* parameter, which is the ratio of a partner effect on an actor effect (*k* = p/a; Kenny and Ledermann 2010). Three values of *k* are noteworthy: a couple pattern (*k* = 1) where the actor and partner effects are equal in size; an actor‐only pattern (*k* = 0) where the partner effect is zero; and a contrast pattern (*k* = −1) where the actor and partner effects are equal in magnitude but differ in sign (Kenny and Ledermann 2010). Finally, to test for distinguishability of paths, we compared models that constrained path coefficients across gender. As suggested by Kenny et al. (2006), a liberal significance level of 0.20 was used to test for distinguishability.

## 7 Results

### 7.1 Preliminary Analyses

Prior to conducting analyses, variables were examined for potential skew, kurtosis, and normality assumptions. Given that the data violated normality assumptions and some variables were skewed, we chose to use maximum likelihood with robust standard errors (MLR). This was chosen over data transformation, as this method can limit the interpretation of data. Zero‐order correlations were then calculated (Table 2). Given that intimacy and problem‐solving behaviors were not correlated with flooding or attachment dimensions, they were dropped from subsequent analyses. Results of paired‐samples t‐tests indicated gender differences within couples. Women reported higher attachment anxiety (*M* = 46.06, SD = 17.65) than men (*M* = 38.69, *S*D = 14.63), *t*(95) = 3.39, *p* < 0.001, *d* = 0.45 and lower attachment avoidance (*M* = 26.53, SD = 9.94) than men (*M* = 32.50, SD = 14.84), *t*(95) = −3.54, *p* < 0.001, *d* = 0.47. Emotional flooding did not differ between women (*M* = 15.86, SD = 6.79) and men (*M* = 17.08, SD = 6.94), *t*(95) = −1.52, *p* = 0.131, *d* = 0.18. With respect to observed conflict behaviors, women displayed less hostility (*M* = 2.82, SD = 2.30) than men (*M* = 5.65, SD = 4.25), *t*(95) = −11.89, *p* < 0.001, *d* = 0.50, and less withdrawal (*M* = 4.17, SD = 2.20) than men (*M* = 4.46, SD = 2.41), *t*(95) = −4.98, *p* < 0.001, *d* = 0.21.

**Table 2:** Correlations, means, and standard deviations for all study variables.

Variable	1	2	3	4	5	M(w)	SD(w)
1. ANX	0.14	0.45***	0.68***	0.22*	0.13	46.06	17.65
2. AVO	0.34***	0.16	0.42***	0.21*	−0.06	26.53	9.94
3. FLO	0.54***	0.37***	0.35***	0.30**	0.10	15.86	6.79
4. HOS	0.20	0.06	0.20	0.91***	0.13	2.82	2.30
5. WDR	−0.06	0.14	0.21*	0.24*	0.85***	4.17	2.20
M(m)	38.69	32.50	17.08	5.65	4.46		
SD(m)	14.63	14.84	6.94	4.25	2.41		

*Note:* Men (m) below the diagonal, women (w) above the diagonal, and interpartner correlations along the diagonal.

Abbreviations: ANX = Attachment anxiety, AVO = attachment avoidance, FLO = emotional flooding, HOS = hostility, and WDR = withdrawal.

### 7.2 APIMeM: Hostility

The indistinguishable model (constraining means, variances, and covariances) did not fit the data well (*χ* ^2^(8) = 109.96, *p* < 0.05; CFI = 0.50; RMSEA = 0.46; and SRMR = 0.24). Freeing these constraints significantly improved model fit, as indicated by a chi‐square difference test comparing the constrained and unconstrained models, *χ* ^2^ = 109.96, *p* < 0.001, indicating gender differences. We then examined *k* effects for the predictor–mediator and mediator–outcome paths and found actor‐only patterns for attachment anxiety and flooding for men (*k*_1_ = 0.216 [−0.164, 0.661]) and women (*k*_2_ = 0.120 [−0.167, 0.248]). This simpler model was chosen, as it did not worsen model fit (*χ* ^2^ = 1.87, *p* = 0.39). Constraining gender resulted in a worse fitting model (*χ* ^2^ = 13.07, *p* = 0.16), indicating gender effects across paths.

**Figure 1:**
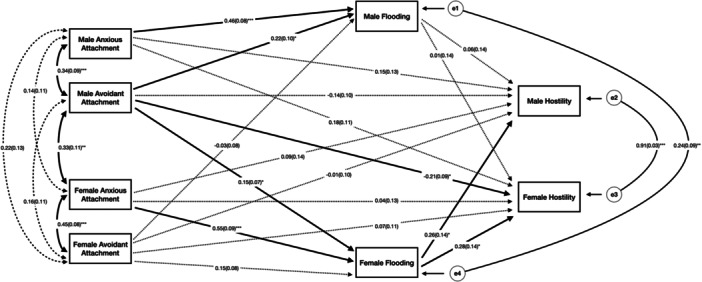
Indirect model of attachment insecurity, emotional flooding, and hostility. *Note:* Full lines indicate significant paths (*p < 0.05, **p < 0.01, and ***p < 0.001) and dotted lines indicate nonsignificant paths.

This final model fit the data well (*χ* ^2^(2) = 1.87, *p* = 0.39; CFI = 1.00; RMSEA = 0.00; SRMR = 0.02). Both men's (*β* = 0.46, *p* < 0.001) and women's (*β* = 0.55, *p* < 0.001) attachment anxiety was positively associated with their own flooding. Men's attachment avoidance was positively associated with their own flooding (*β* = 0.22, *p* < 0.05) and their partner's flooding (*β* = 0.15, *p* < 0.05). Women's flooding was positively associated with their own (*β* = 0.28, *p* < 0.001) and their partner's (*β* = 0.26, *p* < 0.05) hostility. Finally, men's avoidance was negatively associated with their partner's hostility (*β* = −0.21, *p* < 0.05). After bootstrapping, indirect effects were found between women's attachment anxiety and their own hostility through flooding (*β* = 0.15, 95% CI [0.003, 0.317]). This finding suggests that flooding was a potential intermediary variable in the relationship between attachment anxiety and hostility in women. A visual depiction of the results is shown in Figure 1. Standardized estimates with 95% confidence intervals for total, indirect, and direct effects are shown in Supporting Information S1: Table 1.

### 7.3 APIMeM: Withdrawal

The indistinguishable model (constraining means, variances, and covariances) did not fit the data well (*χ* ^2^(8) = 101.17, *p* < 0.05; CFI = 0.69; RMSEA = 0.35; SRMR = 0.20). Freeing these constraints significantly improved model fit, as indicated by a chi‐square difference test comparing the constrained and unconstrained models, *χ* ^2^ = 101.17, *p* < 0.001, indicating distinguishability. We then examined *k* effects for the predictor–mediator and mediator–outcome paths, and found actor‐only patterns for attachment anxiety and flooding for men (*k*_1_ = 0.216 [−0.156, 0.660]) and women (*k*_2_ = 0.120 [−0.170, 0.431]). This simpler model was chosen, as it did not worsen model fit (*χ* ^2^ = 1.87, *p* = 0.39). Constraining gender resulted in a worse fitting model (*χ* ^2^ = 13.71, *p* = 0.13), indicating gender effects across paths.

**Figure 2:**
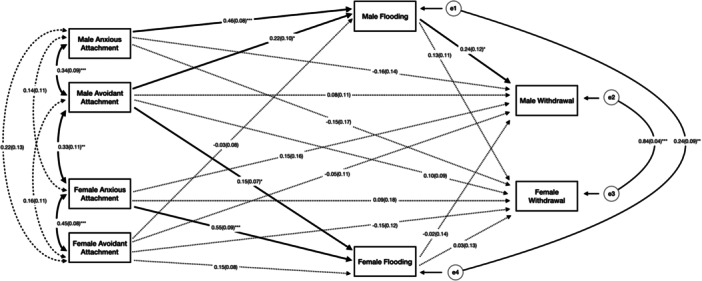
Indirect model of attachment insecurity, emotional flooding, and withdrawal. *Note:* Full lines indicate significant paths (*p < 0.05, **p < 0.01, and ***p < 0.001) and dotted lines indicate nonsignificant paths.

This final model fit the data well (*χ* ^2^(2) = 1.87, *p* = 0.39; CFI = 1.00; RMSEA = 0.00; SRMR = 0.02). Both men's (*β* = 0.46, *p* < 0.001) and women's (*β* = 0.55, *p* < 0.001) attachment anxiety was positively associated with their own flooding. Men's attachment avoidance was positively associated with their own flooding (*β* = 0.22, *p* < 0.05) and their partner's flooding (*β* = 0.15, *p* < 0.05). Men's flooding was positively associated with their own withdrawal (*β* = 0.24, *p* < 0.05). After bootstrapping, indirect effects were found between men's attachment anxiety and their own withdrawal through flooding (*β* = 0.11, 95% CI [0.004, 0.232]). This finding suggests that emotional flooding was a potential intermediary variable in the relationship between attachment anxiety and withdrawal in men. A visual depiction of the results is shown in Figure 2. Standardized estimates with 95% confidence intervals for total, indirect, and direct effects are shown in Supporting Information S1: Table 2.

## 8 Discussion

The present study examined the complex associations between attachment styles, emotional flooding, and conflict behaviors in a dyadic, multimethod study of couples expecting their first child. We were able to examine the dyadic nature of these constructs, providing evidence of the emotional association that may indirectly account for the relationship between attachment insecurity and observed behaviors during couple conflict. Our findings that emotional flooding was associated with attachment insecurity as well as certain conflict behaviors provide additional support for the intra‐ and interpersonal nature of emotion dysregulation and its implications for couple functioning at the prenatal period.

Attachment anxiety was positively associated with individuals' own flooding. Flooding in response to typical conflicts between partners may be more likely among those with higher attachment anxiety, as they are predisposed to heightened emotional and physiological reactivity, which can challenge emotion regulation and increase reliance on negative behaviors (Mikulincer and Shaver 2007; Taylor et al. 2018). Similarly, men's attachment avoidance was linked to both their own and their partner's flooding, suggesting that attempts to downregulate or deactivate emotions may not fully prevent cognitive overwhelm. In fact, avoidant strategies may inadvertently intensify the partner's emotional response, creating a mismatch in emotional needs and highlighting the interpersonal consequences of attachment‐related regulation strategies (Velotti et al. 2016; Winderheld, 2016).

In line with hypothesis 1, women's attachment anxiety was positively linked with their own hostility through their own emotional flooding. In contrast, men's attachment anxiety, rather than their avoidance, was positively linked with their own withdrawal through their own emotional flooding. This divergence from hypothesis 2 may reflect the different pathways through which insecurity relates to flooding. Whereas avoidance is often characterized by emotional suppression, anxiety is marked by hyperactivation and distress‐intensifying appraisals of conflict (Mikulincer and Shaver 2007). These appraisals may have increased men's susceptibility to flooding, making anxiety, rather than avoidance, the more salient predictor. Heightened emotional arousal, such as flooding, may lead individuals to rely on habitual conflict behaviors, including hostility or withdrawal (Christensen and Heavey 1990). Women's flooding was associated not only with their own hostility but also with their partners' displays of hostility, suggesting that individual emotional overwhelm can spill over into interpersonal conflict dynamics. These findings align with theoretical models emphasizing the role of emotional processes in shaping conflict behavior (Mikulincer and Shaver 2007; Patterson 1982) and offer preliminary support for considering emotion regulation as both an intra‐ and interpersonal process within couples (Dixon‐Gordon et al. 2015). This interpretation is consistent with a growing body of literature linking flooding to destructive conflict patterns. For example, Malik et al. (2020) examined flooding and conflict behaviors in 233 married or cohabiting couples and found that men's and women's flooding were positively associated with their partners' expressed anger during conflict.

For men in the present study, withdrawal may reflect a regulatory response to heightened emotional arousal, consistent with “match and step down” dynamics, in which one partner initially matches the intensity of the other's behavior but subsequently reduces engagement, facilitating de‐escalation within the dyad (Slep et al. 2016). Emotional flooding has also been linked to withdrawal during conflict in men specifically (Malik et al. 2020), suggesting that disengagement may serve as an attempt to manage overwhelming affect. Although temporal sequencing could not be directly examined in the current study, these findings provide preliminary evidence that individuals' susceptibility to emotional flooding may meaningfully shape conflict dynamics within couples.

Our finding that flooding was not associated with positive conflict behaviors may reflect the constructs examined, attachment insecurity and emotional flooding, both of which emphasize dysregulation rather than security or positive coping strategies. The link to negative conflict behaviors is consistent with the fight‐or‐flight nature of flooding and the valence of the measures used. The absence of associations with positive behaviors may also reflect the conflict paradigm itself, which constrains the range of behaviors that can be meaningfully connected to flooding. This lack of finding could be partly due to the poor reliability of the positive behavior factors in the CAIRS, which perhaps reduced the ability to detect associations.

Although the observed associations between attachment insecurity, emotional flooding, and conflict behaviors may not be unique to the prenatal period, they complement a growing body of work suggesting that emotional flooding is a salient interpersonal emotion regulation process in couple conflict, particularly during the prenatal period. Longitudinal research indicates that emotional flooding and conflict behaviors observed during pregnancy have downstream implications; for example, men's flooding and women's hostility during pregnancy have been shown to predict increases in psychological aggression in the postpartum period (Sotskova et al. 2015). Similarly, attachment has been consistently linked to poorer adaptation across the transition to parenthood, with a partner's attachment anxiety predicting increases in negative conflict dynamics, such as anger, across this period (Kohn et al. 2012). More recently, Lessard et al. (2025) demonstrated that attachment insecurity assessed prenatally predicted declines in relationship satisfaction following childbirth, with conflict engagement serving as an intermediary variable; they also reported direct associations between attachment avoidance and withdrawal behaviors.

Taken together, this literature suggests that the prenatal period represents a particularly informative window for observing attachment activation and interpersonal emotion dysregulation before the additional demands of caregiving emerge. Although the present study cannot speak to developmental trajectories, examining these processes during pregnancy may help identify early risk patterns that have been shown in prior work to shape later relationship functioning, coparenting dynamics, and family adjustment (Simpson and Rholes 2012).

### 8.1 Clinical Implications

The current study highlights how attachment insecurity and emotional flooding contribute to negative conflict behaviors in couples, particularly during the prenatal period. Emotionally Focused Therapy (EFT; Johnson 2019), grounded in adult attachment theory, directly addresses these processes by helping partners identify attachment‐related insecurities, increase emotional awareness, and practice effective regulation strategies before emotional flooding occurs (Greenberg and Johnson 1988). EFT focuses on three central intervention targets: cycle de‐escalation, withdrawer re‐engagement, and pursuer softening (Johnson 2019). Central to this approach is identifying demand–withdraw patterns of conflict, in which one partner escalates engagement to meet attachment needs while the other disengages to prevent emotional arousal. As the pursuing partner intensifies emotional expression, the withdrawing partner may become overwhelmed, increasing the likelihood of emotional flooding and further entrenching the cycle.

By restructuring these interactional patterns and encouraging partners to act counter to their habitual responses (e.g., supporting withdrawer re‐engagement and pursuer softening), EFT aims to reduce conflict escalation, strengthen emotional bonds, and promote more effective emotion regulation (Johnson 2019). In this way, EFT may indirectly reduce emotional flooding by increasing a partner's capacity to tolerate distress and remain emotionally engaged during conflict. Supporting this interpretation, Huerta and colleagues (2023) identified gendered demand–withdraw patterns in couples, with women more likely to occupy the demanding role and men more likely to withdraw, aligning closely with the hostility and withdrawal patterns observed in the present study. Additionally, higher emotional intelligence has been shown to buffer against emotional flooding (Berenguer‐Soler et al. 2023), suggesting that interventions that enhance emotional awareness and regulation may mitigate the interpersonal processes identified here.

Although couple interventions often target verbal processing of emotionally laden experiences such as conflict, nonverbal approaches may offer an additional pathway for improving conflict regulation. One such approach is the use of affiliative touch (Conradi et al. 2020). Experimental work has shown that handholding during conflict discussion can reduce physiological reactivity (e.g., heart rate), a process closely aligned with emotional flooding. Similar effects have been observed following conflict, suggesting that touch may facilitate more efficient physiological recovery. More broadly, touch has been associated with reductions in subjective stress and improvements in communication behaviors during couple interactions (Jakubiak and Feeney 2019). Within EFT, nonverbal cues such as touch are often used to promote safety, co‐regulation, and emotional engagement (Johnson 2019). Together, these findings suggest that incorporating touch into couple interventions may provide a direct and accessible strategy for reducing emotional reactivity and supporting more adaptive conflict processes.

The prenatal period represents a critical window for intervention, as heightened conflict during pregnancy can set the stage for persistent relational difficulties after childbirth. Despite this, most psychological interventions for first‐time parents focus on mothers or mother–infant dyads, with limited attention to the couple (McHale and Negrini 2018). Incorporating both partners in couples‐focused prenatal interventions has been shown to reduce conflict, improve communication, and yield lasting benefits for child outcomes (Doss et al. 2020; Feinberg et al. 2016; Shapiro et al. 2015). By explicitly addressing attachment insecurity, emotional flooding, and demand–withdraw dynamics during pregnancy, clinicians can equip couples with strategies to navigate conflict more adaptively, potentially reducing the escalation of hostility and withdrawal, and buffering families against longer‐term relational and developmental risks.

### 8.2 Limitations and Future Directions

Although this study provides an understanding of the emotional mechanisms connecting attachment insecurity to conflict behaviors in prenatal couples, it is not without limitations. First, the cross‐sectional design precludes conclusions about temporal or causal processes. Emotional flooding should therefore be interpreted as a general dispositional tendency associated with conflict behaviors, rather than a definitive mechanism linking attachment to conflict. Second, flooding was measured prior to the observed discussions, reflecting individuals' broader susceptibility rather than moment‐to‐moment emotional reactivity during these interactions. Third, the volunteer sample consisted of relatively affluent, predominantly white, mixed‐sex couples with generally low levels of attachment insecurity and emotional flooding. This limits generalizability to more diverse or higher distress populations and may have constrained detection of indirect or partner effects. Sample size may also have reduced power for partner effects, though prior research suggests that actor effects can be detected in modestly sized dyads (e.g., 100 dyads; Ledermann et al. 2022). Finally, flooding was assessed via self‐report, which captures the cognitive appraisal of conflict aversiveness, but not the physiological underpinnings. Gottman (1993) suggested that avoidant individuals might report feeling regulated, but their physiologies suggest the opposite. This could explain the limited findings of attachment avoidance and flooding relating to conflict behaviors in the current study. Although physiological indicators are important, self‐reports capture additional facets of emotion (Bradley & Lang, 2000). Research has found that men are more attuned to their own arousal and negative affect than women (Levenson et al. 1994), making self‐reported flooding a useful lens for examining links between attachment and conflict behaviors.

Given our preliminary findings that emotional flooding relates to both intra‐ and interpersonal regulation, future research should explore these constructs longitudinally to clarify their temporal dynamics. Such studies could investigate how conflict behaviors may perpetuate emotional flooding and activate the attachment system over time. Extending research into the postpartum period, using multi‐method approaches that combine psychophysiological, observational, and self‐report measures could clarify whether prenatal flooding predicts coparenting quality and child outcomes.

## 9 Conclusion

The study provides a glimpse into the complex dynamics of attachment insecurity, emotional flooding, and their associations with specific conflict behaviors, including hostility and withdrawal, in couples expecting their first child. The dyadic design allowed us to examine how partners' attachment styles and propensity to become emotionally flooded relate to conflict dynamics. These findings suggest that increasing individuals' ability to effectively regulate emotions during conflict may decrease aversive behaviors in couples, especially in those with attachment vulnerabilities.

## Supporting information


**Table S1:** Standardized estimates and bootstrap confidence intervals of total, indirect, and direct effects with hostility as the outcome.
**Table S2:** Standardized estimates and bootstrap confidence intervals of total, indirect, and direct effects with withdrawal as the outcome.

## Data Availability

The data that support the findings of this study are available on request from the corresponding author. The data are not publicly available due to privacy or ethical restrictions.
