# Supplementary material for: Understanding the Associations Between Attachment Insecurity, Emotional Flooding, and Conflict Behaviors in Prenatal Couples
Source: J Marital Fam Ther. 2026 May 6;52:e70142. doi: 10.1111/jmft.70142 (PMC13150407; doi:10.1111/jmft.70142)
Supplement: Supplementary file 1 — Table S1: Standardized estimates and bootstrap confidence intervals of total, indirect, and direct effects with hostility as the outcome. Table S2: Standardized estimates and bootstrap confidence intervals of total, indirect, and direct effects with withdrawal as the outcome. [file JMFT-52-0-s001.docx]

**Supplemental Table 1**

*Standardized estimates and bootstrap confidence intervals of total, indirect, and direct effects with hostility as the outcome*

| Effects | β | *95% CI* |
| --- | --- | --- |
| Men’s Anxiety 🡪 Men’s Hostility |  |  |
| Total effect | 0.174 | [-0.050, 0.398] |
| Indirect Effect | 0.029 | [-0.101, 0.163] |
| Direct Effect | 0.146 | [-0.099, 0.389] |
| Women’s Anxiety 🡪 Women’s Hostility |  |  |
| Total Effect | 0.219 | [0.014, 0.426] |
| Indirect Effect | 0.152 | [0.003, 0.317] |
| Direct Effect | 0.041 | [-0.221, 0.306] |
| Men’s Avoidance 🡪 Men’s Hostility |  |  |
| Total Effect | -0.085 | [-0.277, 0.105] |
| Total Indirect Effect | 0.053 | [-0.023, 0.157] |
| Men’s avoidance, Men’s flooding, Men’s hostility | 0.014 | [-0.053, 0.090] |
| Men’s avoidance, Women’s flooding, Men’s hostility | 0.039 | [-0.004, 0.120] |
| Direct Effect | -0.138 | [-0.326, 0.048] |
| Women’s Avoidance 🡪 Women’s Hostility |  |  |
| Total Effect | 0.108 | [-0.094, 0.306] |
| Total Indirect Effect | 0.041 | [-0.018, 0.120] |
| Women’s avoidance, Women’s flooding, Women’s hostility | 0.041 | [-0.008, 0.118] |
| Women’s avoidance, Men’s flooding, Women’s hostility | 0.000 | [-0.025, 0.023] |
| Direct Effect | 0.067 | [-0.142, 0.273] |
| Men’s Anxiety 🡪 Women’s Hostility |  |  |
| Total effect | 0.186 | [-0.023, 0.393] |
| Indirect Effect | 0.004 | [-0.128, 0.136] |
| Direct Effect | 0.182 | [-0.036, 0.402] |
| Women’s Anxiety 🡪 Men’s Hostility |  |  |
| Total Effect | 0.138 | [-0.085, 0.363] |
| Indirect Effect | 0.144 | [-0.005, 0.301] |
| Direct Effect | -0.006 | [-0.197, 0.183] |
| Men’s Avoidance 🡪 Women’s Hostility |  |  |
| Total Effect | -0.166 | [-0.349, 0.018] |
| Total Indirect Effect | 0.043 | [-0.034, 0.136] |
| Men’s avoidance, Men’s flooding, Women’s hostility | 0.002 | [-0.067, 0.072] |
| Men’s avoidance, Women’s flooding, Women’s hostility | 0.041 | [-0.004, 0.118] |
| Direct Effect | -0.209 | [-0.378, -0.037] |
| Women’s Avoidance 🡪 Men’s Hostility |  |  |
| Total Effect | 0.032 | [-0.157, 0.217] |
| Total Indirect Effect | 0.038 | [-0.026, 0.116] |
| Women’s avoidance, Women’s flooding, Men’s hostility | 0.039 | [-0.008, 0.114] |
| Women’s avoidance, Men’s flooding, Men’s hostility | -0.002 | [-0.033, 0.020] |
| Direct Effect | -0.006 | [-0.197, 0.183] |

*Note*. Analyses based on 5,000 bootstrapped draws. β = standardized estimates; *95% CI = 95% Confidence Interval*.

**Supplemental Table 2**

*Standardized estimates and bootstrap confidence intervals of total, indirect, and direct effects with withdrawal as the outcome*

| Effects | β | *95% CI* |
| --- | --- | --- |
| Men’s Anxiety 🡪 Men’s Withdrawal |  |  |
| Total Effect | -0.048 | [-0.278, 0.185] |
| Indirect Effect | 0.110 | [0.004, 0.232] |
| Direct Effect | -0.158 | [-0.437, 0.122] |
| Women’s Anxiety 🡪 Women’s Withdrawal |  |  |
| Total Effect | -0.129 | [-0.437, 0.122] |
| Indirect Effect | 0.018 | [-0.124, 0.161] |
| Direct Effect | 0.087 | [-0.252, 0.433] |
| Men’s Avoidance 🡪 Men’s Withdrawal |  |  |
| Total Effect | 0.135 | [-0.079, 0.351] |
| Total Indirect Effect | 0.051 | [-0.017, 0.144] |
| Men’s avoidance, Men’s flooding, Men’s withdrawal | 0.053 | [-0.001, 0.138] |
| Men’s avoidance, Women’s flooding, Men’s withdrawal | -0.003 | [-0.046, 0.047] |
| Direct Effect | 0.084 | [-0.134, 0.298] |
| Women’s Avoidance 🡪 Women’s Withdrawal |  |  |
| Total Effect | -0.145 | [-0.394, 0.099] |
| Total Indirect Effect | 0.002 | [-0.050, 0.054] |
| Women’s avoidance, Women’s flooding, Women’s withdrawal | 0.005 | [-0.041, 0.052] |
| Women’s avoidance, Men’s flooding, Women’s withdrawal | -0.003 | [-0.034, 0.021] |
| Direct Effect | -0.147 | [-0.383, 0.095] |
| Men’s Anxiety 🡪 Women’s Withdrawal |  |  |
| Total Effect | -0.090 | [-0.374, 0.199] |
| Indirect Effect | 0.060 | [-0.035, 0.168] |
| Direct Effect | -0.150 | [-0.477, 0.184] |
| Women’s Anxiety 🡪 Men’s Withdrawal |  |  |
| Total Effect | -0.056 | [-0.302, 0.192] |
| Indirect Effect | -0.010 | [-0.162, 0.140] |
| Direct Effect | -0.047 | [-0.266, 0.173] |
| Men’s Avoidance 🡪 Women’s Withdrawal |  |  |
| Total Effect | 0.134 | [-0.048, 0.317] |
| Total Indirect Effect | 0.034 | [-0.026, 0.112] |
| Men’s avoidance, Men’s flooding, Women’s withdrawal | 0.029 | [-0.017, 0.093] |
| Men’s avoidance, Women’s flooding, Women’s withdrawal | 0.005 | [-0.036, 0.053] |
| Direct Effect | 0.100 | [-0.076, 0.272] |
| Women’s Avoidance 🡪 Men’s Withdrawal |  |  |
| Total Effect | -0.056 | [-0.280, 0.172] |
| Total Indirect Effect | -0.009 | [-0.074, 0.048] |
| Women’s avoidance, Women’s flooding, Men’s withdrawal | -0.003 | [-0.059, 0.043] |
| Women’s avoidance, Men’s flooding, Men’s withdrawal | -0.006 | [-0.049, 0.035] |
| Direct Effect | -0.047 | [-0.266, 0.173] |

*Note*. Analyses based on 5,000 bootstrapped draws. β = standardized estimates; *95% CI = 95% Confidence Interval*.
